# Modeling the glutamate–glutamine neurotransmitter cycle

**DOI:** 10.3389/fnene.2013.00001

**Published:** 2013-01-28

**Authors:** Jun Shen

**Affiliations:** Molecular Imaging Branch, National Institute of Mental HealthBethesda, MD, USA

**Keywords:** glutamate, glutamine, magnetic resonance spectroscopy, glucose metabolism, CNS, metabolic modeling, acetate

## Abstract

Glutamate is the principal excitatory neurotransmitter in brain. Although it is rapidly synthesized from glucose in neural tissues the biochemical processes for replenishing the neurotransmitter glutamate after glutamate release involve the glutamate–glutamine cycle. Numerous *in vivo* ^13^C magnetic resonance spectroscopy (MRS) experiments since 1994 by different laboratories have consistently concluded: (1) the glutamate–glutamine cycle is a major metabolic pathway with a flux rate substantially greater than those suggested by early studies of cell cultures and brain slices; (2) the glutamate–glutamine cycle is coupled to a large portion of the total energy demand of brain function. The dual roles of glutamate as the principal neurotransmitter in the CNS and as a key metabolite linking carbon and nitrogen metabolism make it possible to probe glutamate neurotransmitter cycling using MRS by measuring the labeling kinetics of glutamate and glutamine. At the same time, comparing to non-amino acid neurotransmitters, the added complexity makes it more challenging to quantitatively separate neurotransmission events from metabolism. Over the past few years our understanding of the neuronal-astroglial two-compartment metabolic model of the glutamate–glutamine cycle has been greatly advanced. In particular, the importance of isotopic dilution of glutamine in determining the glutamate–glutamine cycling rate using [1−^13^C] or [1,6-^13^C_2_] glucose has been demonstrated and reproduced by different laboratories. In this article, recent developments in the two-compartment modeling of the glutamate–glutamine cycle are reviewed. In particular, the effects of isotopic dilution of glutamine on various labeling strategies for determining the glutamate–glutamine cycling rate are analyzed. Experimental strategies for measuring the glutamate–glutamine cycling flux that are insensitive to isotopic dilution of glutamine are also suggested.

## Introduction

Glutamate is the principal excitatory neurotransmitter in brain. At low concentrations it excites virtually all neurons in the CNS. Excessive activation of glutamate receptors by glutamate can result in a number of pathological conditions and can lead to cell death. As a zwitterionic molecule glutamate cannot diffuse across cell membranes. It is well understood that glutamate uptake plays important roles in regulating the extracellular concentration of glutamate in the brain. Both *in vivo* and *in vitro* studies have indicated that glutamate released by neurons is rapidly taken up by astroglial cells, via high-affinity Na^+^-dependent glutamate transporters. Evidence from studies using antisense mRNA to selectively knock down neuronal and astroglial transporters, as well as direct measurements of glutamate-gated ionic currents, support the hypothesis that almost all released glutamate is taken up by astroglia in the cerebral cortex (Rothstein, 1996; Bergles et al., 1999). Subsequently, glutamate is either converted into glutamine by glutamine synthetase, which is exclusively localized in glial cells (Martinez-Hernandez et al., 1977), or oxidized by assimilation into the Krebs cycle located in the mitochondria of astroglial cells. Although glutamate is rapidly synthesized from glucose in neural tissues the biochemical processes for replenishing the neurotransmitter glutamate after glutamate release involve the glutamate–glutamine cycle (Cerdán et al., 1990; Erecińska and Silver, 1990). Glutamine, formed by amidization of glutamate, is readily discharged from astroglial cells by facilitated diffusion via Na^+^ and H^+^-coupled, electroneutral systems-N transporters. Glutamine readily enters nerve terminals mainly by electrogenic systems-A transporters (Chaudhry et al., 2002). There glutaminase converts it back into glutamate which can be again used for neuronal transmission or assimilated into the neuronal Krebs cycle.

The existence of this glutamate–glutamine cycle was initially proposed based on the multiple findings: (1) isolated nerve terminals contain the majority of tissue content of glutaminase but no glutamine synthetase, the latter is found to be exclusively located in glial cells (Martinez-Hernandez et al., 1977; Hertz, 1979); (2) biochemical and autoradiographic studies clearly demonstrated that glutamate is selectively accumulated by glial cells and rapidly converted into glutamine; (3) by comparison, glutamine preferentially enters neurons where it is converted in large proportions into glutamate (Duce et al., 1983). The glutamate–glutamine cycling pathway between neurons and astroglia has been studied extensively *in vivo*, in cell culture, and in brain slices using isotope tracers (e.g., Shank et al., 1993; Lapidot and Gopher, 1994). Despite the large amount of evidence for its existence, early kinetic studies considered that the glutamate–glutamine cycling flux is small and makes only a minor contribution to brain energy metabolism. Consistent with this early notion, the fraction of glutamate participating in the glutamate–glutamine cycle was considered small, leading to the conceptualization of a neurotransmitter glutamate pool and a separate metabolic glutamate pool (Erecińska and Silver, 1990). This compartmentalization of neuronal glutamate is supported by the experimental findings of a low rate of label incorporation in cell cultures and in non-electrically stimulated brain slices from various labeled precursors (e.g., Badar-Goffer et al., 1992).

The initial detection of glucose metabolism using *in vivo* ^13^C magnetic resonance spectroscopy (MRS) found rapid and significant labeling of glutamine (Gruetter et al., 1994), which is predominantly located in the astroglial cells. This *in vivo* evidence suggests rapid transfer of ^13^C labels from the predominantly neuronal glutamate compartment to the predominantly astroglial glutamine compartment. Subsequent studies on human and animal brains using refined MRS techniques and various ^13^C-labeled substrates have consistently contradicted the concept of a small, metabolically inactive neurotransmitter glutamate pool (for a recent review, see, for example, Rothman et al., 2011). Using [1-^13^C] glucose or [1,6-^13^C_2_] glucose infusion, which predominantly labels neuronal glutamate due to the high energy demand by neurons, rapid and significant labeling of glutamine C2–C4 has been consistently reproduced by all studies employing either direct ^13^C detection or the more sensitive indirect proton detection. Using [2-^13^C] glucose or [2,5-^13^C_2_] glucose and direct ^13^C detection of carboxyl/amide carbons the similar labeling of glutamine C5 was also found (Li et al., 2009). The findings of rapid labeling of glutamine by ^13^C-labeled glucose that is predominantly metabolized in the neuronal compartment are corroborated by ^15^N MRS studies of ^15^NH_3_ labeling of glutamine in a hyperammonemia model (Shen et al., 1998). Preferential labeling of the predominantly astroglial glutamine can be achieved by utilizing either the glia-specific anaplerotic pathway or the preference for acetate by glial cells. Using [2-^13^C] glucose or [2,5-^13^C_2_] glucose infusion, rapid transfer of ^13^C labels from glutamine C3 and C2 to glutamate C3 and C2 was observed (Sibson et al., 2001). Similarly, when the glia-specific substrate acetate is infused glutamine is labeled first followed by glutamate reflecting the transfer of ^13^C labels from astroglia to neurons (Lebon et al., 2002; Deelchand et al., 2009). The numerous *in vivo* ^13^C- and ^15^N MRS studies since 1994 by different laboratories have consistently concluded: (1) glutamate–glutamine cycling is a major metabolic pathway with a flux rate substantially greater than those suggested by early studies of cell cultures and brain slices; (2) the glutamate–glutamine cycle is coupled to a large portion of the total energy demand of brain function (Rothman et al., 2011).

Extracting quantitative flux rates from any metabolic study requires modeling, which, historically, has led to controversial results with few exceptions. The dual roles of glutamate as the principal neurotransmitter in the CNS and as a key metabolite linking carbon and nitrogen metabolism make it possible to probe glutamate neurotransmitter cycling using MRS by measuring the labeling kinetics of glutamate and glutamine. At the same time, comparing to non-amino acid neurotransmitters, the added complexity makes it more challenging to quantitatively separate neurotransmission events from metabolism. Although the findings from *in vivo* MRS studies are in agreement that the glutamate–glutamine cycle is a major metabolic pathway flux reflecting presynaptic glutamate release, significantly different cycling rates have been reported by different laboratories for the same or similar physiological conditions (Gruetter et al., 2001; Shen and Rothman, 2002; Shestov et al., 2007; Rothman et al., 2011). Since the MRS measures total glutamate and glutamine the absolute rates of ^13^C labeling kinetics depend on the rate of the exchange of TCA cycle intermediates across the inner mitochondrial membrane (V_x_). Evidence for both a fast exchange (i.e., V_x_ ≫ V_TCA_; Mason et al., 1995; de Graaf et al., 2004; Patel et al., 2004; Yang et al., 2009) and a slow exchange (i.e., V_x_ ≈ V_TCA_; Gruetter et al., 2001; Berkich et al., 2005) has been presented in the literature. Part of the controversy surrounding the magnitude of V_cyc_ can be traced to whether V_x_ ≫ V_TCA_ or V_x_ ≈ V_TCA_ is used in the two-compartment model.

In addition to the V_x_ issues, a recent study claimed that, with the signal-to-noise ratio achievable by *in vivo* MRS, it is very difficult, if possible, to quantitatively determine the glutamate–glutamine cycling rate at a useful precision (Shestov et al., 2007). Over the past few years our understanding of the neuronal-astroglial two-compartment metabolic model of the glutamate–glutamine cycle has been greatly advanced. In particular, the importance of isotopic dilution of glutamine in determining the glutamate–glutamine cycling rate using the glutamate-to-glutamine label transfer has been demonstrated (Shen et al., 2009) and reproduced (Duarte et al., 2011; Shestov et al., 2012). Recent publications from different laboratories have shown a clear consensus that glutamate–glutamine cycling rate can be determined using *in vivo* MRS with high precision (Boumezbeur et al., 2010a; Duarte et al., 2011; Rothman et al., 2011; Shestov et al., 2012). Most recently, by analyzing ^13^C MRS experiments performed by several laboratories in both rats and humans which measure both neuronal energy consumption and glutamate–glutamine cycling, the Yale group has shown that different studies (both human and animal studies) are highly consistent in terms of the relationship between neuronal energy consumption and the rate of the glutamate–glutamine cycle, which shows that ~80% of the resting energy consumption in the awake brain is coupled to neuronal activity (Hyder and Rothman, 2012). In this article, we first give an overview of the two-compartment glutamate–glutamine cycle model. To highlight the main features and implications of the glutamate–glutamine cycle, a simplified two-compartment model that captures the major ^13^C label flows is analyzed in detail. Recent developments in two-compartment modeling of the glutamate–glutamine cycle are reviewed. In particular, the effects of isotopic dilution of glutamine on various labeling strategies for determining the glutamate–glutamine cycle are analyzed in detail. Experimental strategies for measuring the absolute rate of the glutamate–glutamine cycling flux that are insensitive to isotopic dilution of glutamine are also suggested.

## The two-compartment model of the glutamate–glutamine cycle

**Figure 1:**
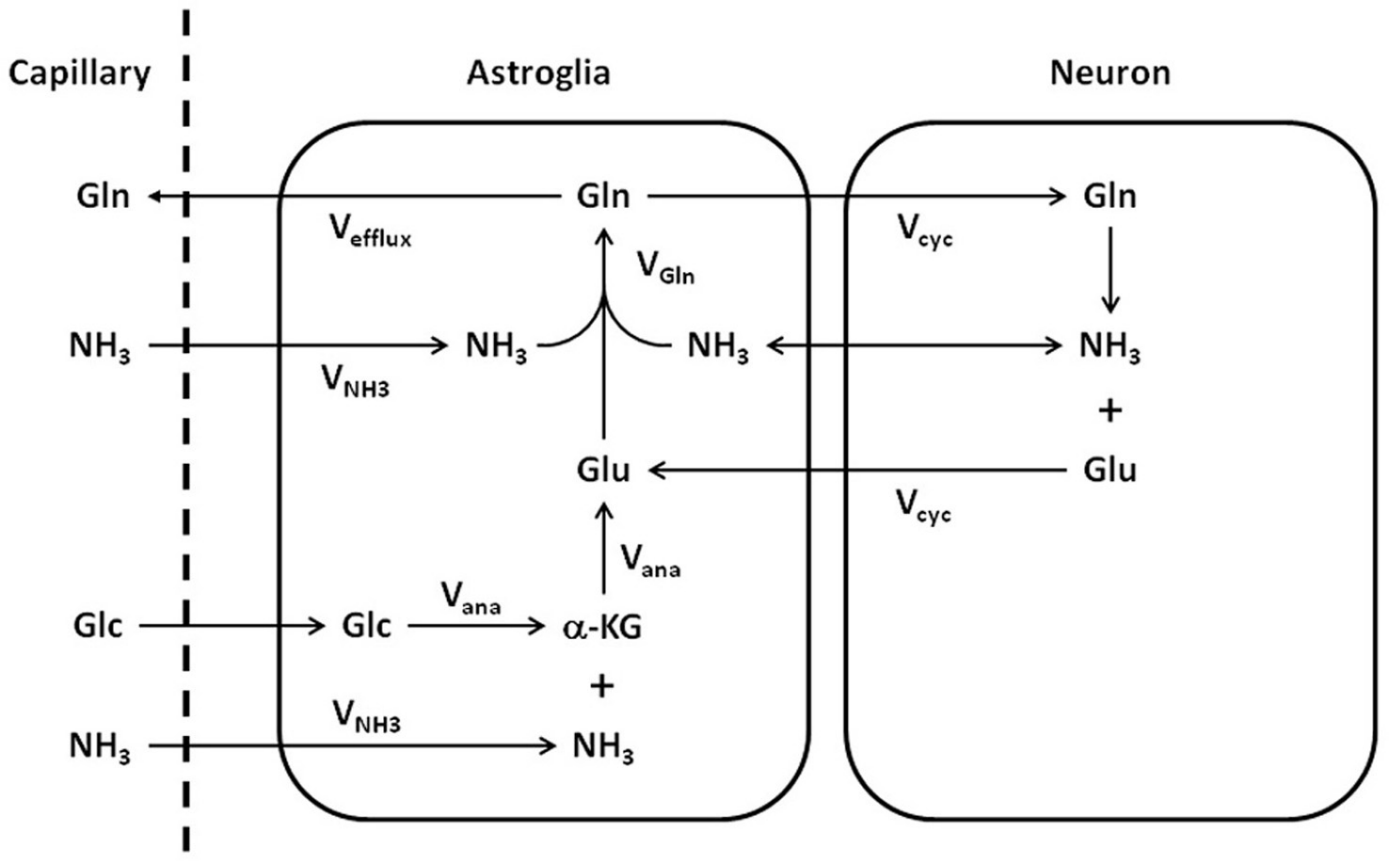
**Schematic representation of the glutamate–glutamine cycle between neurons and astroglia and the ammonia detoxification pathway (adapted from Sibson et al., 1997).** Released neurotransmitter glutamate (Glu) is transported from the synaptic cleft to surrounding astroglial end processes. Once in the astroglia, glutamate is converted to glutamine (Gln) by glutamine synthetase. Glutamine is released by the astroglia, transported into the neurons, and converted to glutamate by glutaminase, which completes the cycle. The net rate of glutamine synthesis reflects both glutamate–glutamine neurotransmitter cycling (V_cyc_) and anaplerosis (V_ana_). NH_3_, ammonia; V_NH3_, ammonia fixation; V_gln_, glutamine synthesis; Glc, glucose; α-KG, α-ketoglutarate.

Important metabolic couplings exist among various cells through the use of common substrates and the exchange of several metabolic intermediates such as glutamate, glutamine, and γ-aminobutyric acid (GABA) (Erecińska and Silver, 1990; Cerdán et al., 2006). Because glutamate also acts as the major excitatory neurotransmitter in the CNS, the neurotransmission of glutamate is intimately related to the metabolism of glutamate and glutamine. To quantitatively model glutamate metabolism and its neurotransmission in the CNS, Sibson et al. (1997) proposed a quantitative two-compartment model describing the glutamate–glutamine cycle between neurons and astroglia (Figure 1). In this model, glutamate released by glutamatergic neurons into the synaptic cleft is taken up by surrounding astroglia and converted into its inactive form glutamine by the glia-specific glutamine synthetase. The abundant expression of high capacity glutamate transporters on glial cell membrane ensures that the extracellular glutamate concentration is kept very low in normal brain to avoid excitotoxicity. To replenish the neuronal carbon lost to astroglia, resulting from synaptic glutamate release, glutamine is released by astroglia and recycled back to neurons where it is hydrolyzed into glutamate by glutaminase. The model shown in Figure 1 imposes mass balance constraints for all carbon and nitrogen fluxes across the blood–brain barrier and between neurons and astroglia at metabolic steady state. At metabolic steady state, the rate of glutamate release by nerve terminals, the subsequent uptake and glutamine synthesis by and in astroglial cells, as well as glutamine uptake and conversion into glutamate in neurons are equal (V_cycle_). The rate of glutamine synthesis (V_gln_) is the sum of the rate of anaplerotic de novo glutamine synthesis in the astroglia (V_ana_), the glial-specific process in which CO_2_ and pyruvate derived from glucose are converted into oxaloacetate by pyruvate carboxylase (Berl et al., 1963), and the rate of the glutamate–glutamine cycle between neurons and astroglia:

(1) $${\text{V}}_{\text{Gln}}={\text{V}}_{\text{cyc}}+{\text{V}}_{\text{ana}}$$

In addition, the Sibson et al. model connects Vana to the net uptake of anaplerotic precursors from the blood. At the metabolic steady state, glutamine efflux (V_efflux_) is balanced by glutamine de novo synthesis via anaplerosis (V_ana_):

(2) $${\text{V}}_{\text{ana}}={\text{V}}_{\text{efflux}}$$

Following CO_2_ fixation, oxaloacetate is converted into glutamate either by ammonia fixation (V_NH3_) or transamination. Glial glutamate is subsequently converted to glutamine by glutamine synthetase.

**Figure 2:**
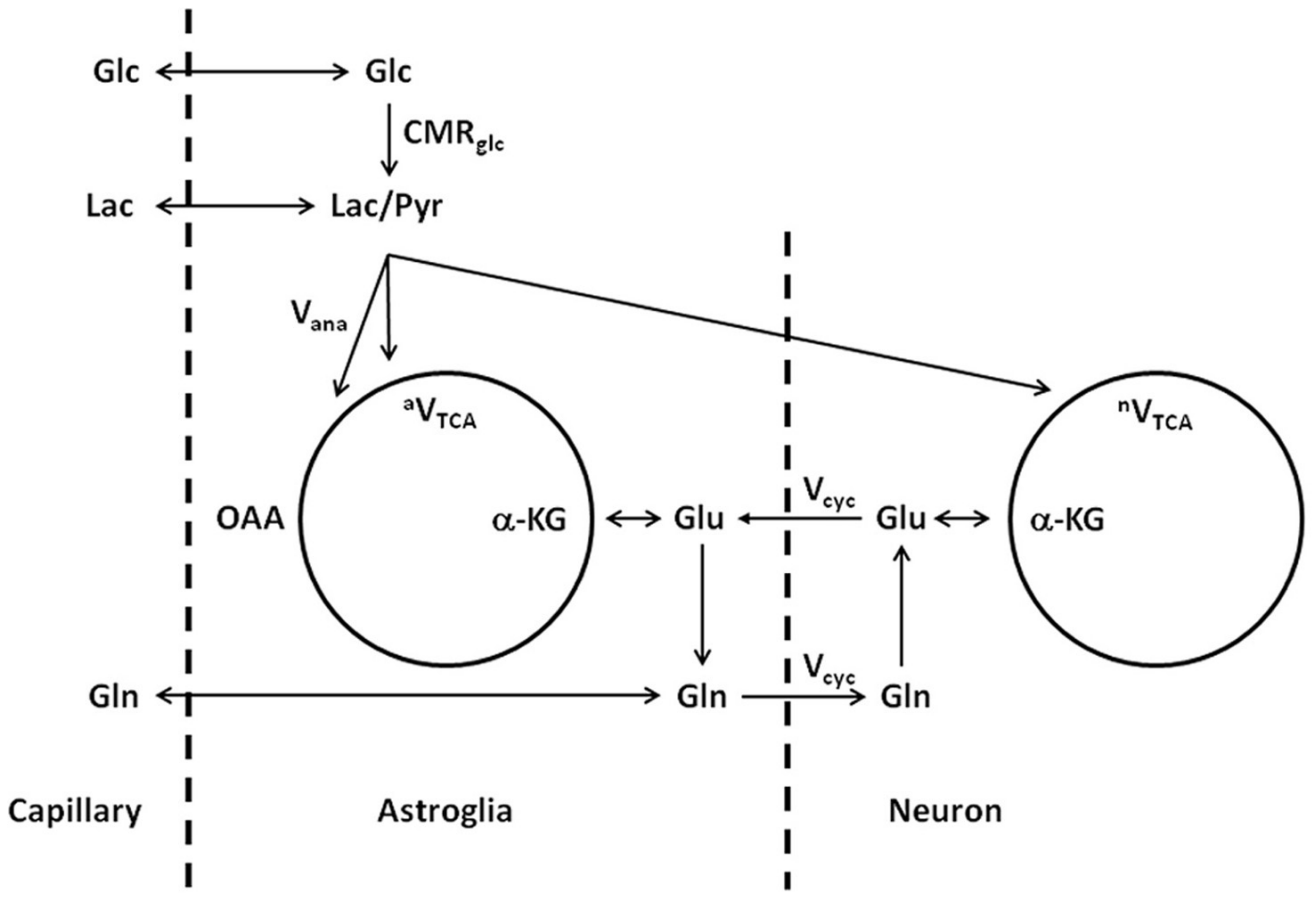
**Schematic illustration of the glutamate–glutamine cycle between neurons and astroglia and glucose metabolism (adapted from Shen et al., 1999).** Released neurotransmitter glutamate is transported from the synaptic cleft by surrounding astroglial end processes. In astroglia, glutamate is converted into glutamine by glutamine synthetase. Glutamine is then released by the astroglia, transported into the neurons, and converted back into glutamate by glutaminase, which completes the cycle. Glc, glucose; Pyr/Lac, pyruvate/lactate; OAA, oxaloacetate; α-KG, α-ketoglutarate; Glu, glutamate; Gln, glutamine; CMRglc, cerebral metabolic rate of glucose utilization; V_ana_, anaplerotic flux for de novo synthesis of oxaloacetate; ^a^V_TCA_, astroglial tricarboxylic acid cycle flux; V_cyc_, glutamate–glutamine cycling flux; ^n^V_TCA_, neuronal tricarboxylic acid cycle flux.

The above model describes the same glutamate–glutamine cycle proposed decades ago (Hertz, 1979; Erecińska and Silver, 1990). The key difference lies in the interpretation of the rapid glutamine labeling observed using *in vivo* MRS. To illustrate the labeling of glutamate and glutamine, a different illustration of the glutamate–glutamine cycle is shown in Figure 2, where the coupling between the glutamate–glutamine cycle and energy metabolism is explicitly shown.

In Figure 2, with infusion of ^13^C-labeled glucose ^13^C labels are mainly incorporated into neuronal glutamate first, accompanying the intensive metabolic activities in neurons. Although seldom emphasized in the literature, it is important to point out that the labeling of glutamate and glutamine is on a very slow time scale when compared to the rapid vesicular release of glutamate and its uptake by astroglia. Experimentally, the *in vivo* turnover time constant of glutamate and glutamine is roughly one hour at resting state in the human brain. At this time scale, there is essentially no distinction between neurotransmitter pool and metabolic pool of glutamate as far as the labeling of the glutamate and glutamine are concerned. It is not surprising that, using *in vivo* microdialysis and mass spectrometry to determine the labeling of extracellular glutamate and glutamine, neuronal glutamate (through glutamate–glutamine cycling) was found to be the precursor for 80–90% of astroglial glutamine synthesis (Kanamori et al., 2003). Therefore, on the time scale of glutamate and glutamine turnover the experimental observation of rapid labeling of glutamine, which is predominantly located in astroglial cells, lead to the logical conclusion that neuronal glutamate is the main metabolic precursor of astroglial glutamine via the glutamate–glutamine cycling flux. In a subsequent analysis of energy cost associated with the glutamate–glutamine cycle Sibson et al. (1998) noticed that glutamate uptake into astroglial cells and its subsequent conversion to glutamine there costs two ATPs. This energy cost is matched by the number of ATPs produced by astroglial glycolysis. By extending the Magistretti hypothesis (Pellerin and Magistretti, 1994) that atroglial glycolysis is coupled to glucose oxidative in neurons Sibson et al. proposed that, although the glutamate–glutamine cycling uses two ATPs in the astrocytic portion of the cycle, the full cycle itself is coupled to the production of a 36 ATPs per glutamate release which are thought to be mostly used for other activities in the brain.

**Figure 3:**
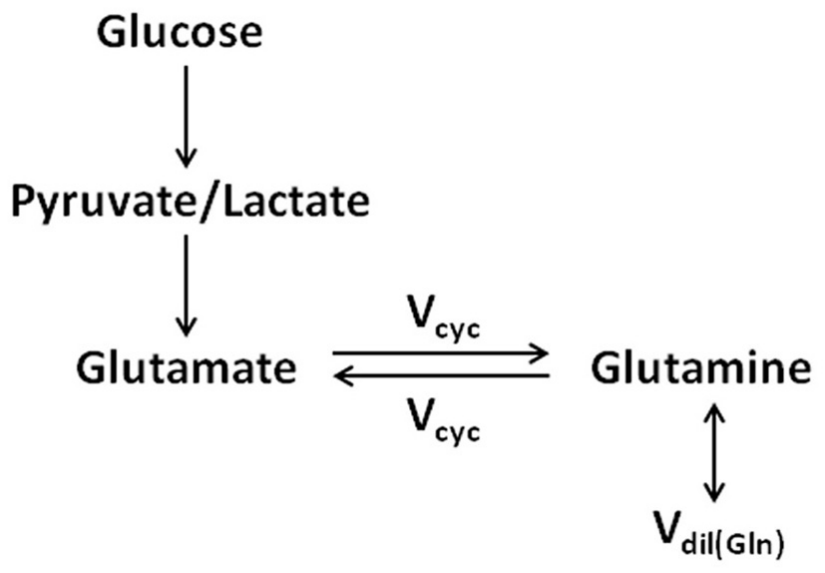
**The simplified two-compartment model in which all ^13^C labels flow from pyruvate/lactate to neuronal glutamate, which is in exchange with astroglial glutamine (adapted from Shen et al., 2009).** This simplified model represents the dominant metabolic pathways for the cycling of ^13^C labels when [1-^13^C] or [1,6-^13^C_2_] glucose infusion is used to introduce exogenous ^13^C labels into the brain. As captured by the simplified model, most ^13^C labels flow from pyruvate C3 to neuronal glutamate C4 and then to astroglial glutamine C4. The astroglial dilution flux was explicitly added at the level of glutamine.

The identification of neuronal glutamate as the main metabolic precursor of astroglial glutamine via the glutamate–glutamine cycling flux leads to a simplified (while conceptually clearer) model of the glutamate–glutamine cycle (see Figure 3), which captures the main feature of intercompartmental trafficking of glutamate and glutamine molecules (Shen et al., 2009). Note that in Figure 3, the astroglial dilution flux, which will be elaborated later, was explicitly added at the level of glutamine. This simplified model represents the dominant metabolic relationship between neuronal glutamate and astroglial glutamine in the brain. That is, when [1-^13^C] or [1,6-^13^C_2_] glucose infusion is used to introduce exogenous ^13^C labels into the brain, most labels flow from pyruvate C3 to neuronal glutamate C4 and then to astroglial glutamine C4. To better illustrate the conceptual aspects of modeling of the glutamate–glutamine cycle this simplified model will be repeatedly referred to.

## *In vivo* MRS methods

Both ^13^C and ^15^N MRS methods have been developed and used to determine the glutamate–glutamine cycling rate. ^13^C MRS methods have been applied to both human subjects and animals while ^15^N MRS methods are limited to animal models of hyperammonemia. Only the ^13^C MRS methods are summarized here. Table 1 lists various ^13^C-related MRS techniques for studying the CNS, especially the glutamate–glutamine cycle. They fall into two main categories based on the choice of observed nucleus (^13^C or ^1^H). Generally, ^13^C detection affords more spectral information. In the aliphatic (carboxyl/amide) spectral region, glutamate and glutamine C2–C4 (C1 and C5) carbons can be measured. Proton detection methods (POCE) have higher sensitivity at the expense of more spectral overlap. ^1^H{^13^C} methods that incorporate proton editing are yet to be fully developed.

**Table 1:** **Heteronuclear MRS methods for studying the glutamate–glutamine cycle using infusion of ^13^C-labeled substrates**.

^13^C{^1^H}	Detect aliphatic carbons such as glutamate C4 and glutamine C4; use either carbon excitation or proton excitation and subsequent proton-to-carbon polarization transfer; need high power coherent proton decoupling (de Graaf et al., 2011)
	Detect carboxylic carbons such as glutamate C5 and glutamine C5; use carbon excitation; can use low power stochastic proton decoupling (Li et al., 2009)
^1^H{^13^C}	Detect aliphatic protons such as glutamate H4 and glutamine H4; use proton excitation and proton detection; need high power coherent carbon decoupling (de Graaf et al., 2011)

## Labeling strategies

Accompanying the advancement of *in vivo* MRS technology many methods have been developed for labeling glutamate and glutamine by administering ^13^C-labeled exogenous substrates (e.g., Sibson et al., 1997, 2001; Blüml et al., 2002; Lebon et al., 2002; Deelchand et al., 2009). The most commonly used substrate is D-glucose, which is the primary source of energy for brain metabolism and function under normal physiological conditions. As shown by Figure 3, with infusion of [1-^13^C] or [1,6-^13^C_2_] glucose ^13^C labels are mainly incorporated into neuronal glutamate C4 first. Since neuronal glutamate is the main metabolic precursor of astroglial glutamine via the glutamate–glutamine cycling flux, ^13^C label incorporation into glutamine C4 occurs mainly via the glutamate–glutamine cycling pathway (V_cyc_). The simplified model shown in Figure 3 excludes the contribution to the labeling of glutamine C4 via the internal TCA cycle of the astroglial cells, assumed to be small. Subsequent turns of the TCA cycles move the ^13^C labels into C3, C2, C1 of glutamate and glutamine, and eventually carbon dioxide. ^13^C labels on the C3 and C2 carbons of glutamate and glutamine can be readily detected in the aliphatic carbon region of the spectra and their labeling kinetics may be used to improve the determination of the glutamate–glutamine cycling rate. Since the exact contribution to the labeling of glutamine C4 by oxidative metabolism in astroglial cells is difficult to measure by the use of [1-^13^C] or [1,6-^13^C_2_] glucose alone, alternative labeling strategies for *in vivo* ^13^C MRS have been developed and applied to measuring the glutamate–glutamine cycling rate.

In addition to the main pathway of ^13^C label flow depicted by the simplified model of Figure 3, the astroglial anaplerotic pathway (V_ana_) was explicitly shown in Figures 1 and 2. The primarily astroglial anaplerotic pathway in the CNS offers an alternative route, albeit less effective than V_cyc_, of incorporating ^13^C labels into glutamate and glutamine. By the action of the astroglial enzyme pyruvate carboxylase (Patel, 1974) ^13^C labels originated from [1-^13^C] or [1,6-^13^C_2_] glucose labels pyruvate C3. Through CO_2_ (either ^13^CO_2_ or ^12^CO_2_) fixation the astroglial anaplerotic pathway generates the TCA cycle intermediate [3-^13^C] or [3,4-^13^C_2_] OAA, which through the first turn of the TCA cycle, labels C2 of α-ketoglutarate, followed by C2 of glutamate and glutamine. Label scrambling at fumarate due to potential backward flux from OAA to fumarate will label glutamate and glutamine C3 in the first turn of the TCA cycle as well (Merle et al., 1996; Brekke et al., 2012). Depending on if the acetylCoA that condenses with OAA generated by CO_2_ fixation is labeled or not by the infused [1-^13^C] or [1,6-^13^C_2_] glucose, then either [2-^13^C] glutamine or [2,4-^13^C_2_] glutamine is produced during the first turn of the astroglial TCA cycle. Unfortunately, the weak glutamine ^13^C NMR signal contributed by astroglial anaplerosis is strongly masked by the much larger contribution from neuronal oxidative metabolism and the main route of ^13^C label incorporation into glutamine via the glutamate–glutamine cycle (V_cyc_).

The strong interference from the heavy ^13^C traffic coming from neurons, fortunately, can be more or less eliminated by choosing [2-^13^C] or [2,5-^13^C_2_] glucose instead of [1-^13^C] or [1,6-^13^C_2_] glucose (Badar-Goffer et al., 1992). The primary route of ^13^C labeling from [2-^13^C] or [2,5-^13^C_2_] glucose leads to labeling of pyruvate C2 followed by glutamate and glutamine C5 after the first turn of the TCA cycles. Parenthetically, this fact also prompted the development of carboxyl/amide carbon MRS with low power stochastic proton decoupling (Li et al., 2009). Without the anaplerotic pathway only natural abundance (1.1%) ^13^C signals are expected in the aliphatic carbon spectral region, with the possible exception of minor contributions that may arise from isotopic impurity of the infused glucose, label scrambling via hepatic gluconeogenesis, the pentose phosphate shunt and pyruvate recycling (Cerdán et al., 1990). The major contribution to the aliphatic ^13^C signals therefore comes from the astroglial anaplerotic pathway. Through CO_2_ (either ^13^CO_2_ or ^12^CO_2_) fixation catalyzed by the astroglial enzyme pyruvate carboxylase the astroglial anaplerotic pathway generates the TCA cycle intermediate [2-^13^C] or [2,4-^13^C_2_] OAA. As a result, the ^13^C labeling via the astroglial anaplerotic pathway labels C3 of astroglial glutamate (and glutamine) during the first turn of the astroglial TCA cycle. Since the heavy ^13^C traffic generated inside neurons labels carboxyl/amide carbons the kinetics of ^13^C label flow from astroglial glutamine C3 to neuronal glutamate C3 could be measured if sensitivity of the method is sufficient. An independent measurement of the more elusive astroglial TCA cycle rate could also be achieved (Sibson et al., 2001).

The sensitivity of the above approach is ultimately limited by the much smaller V_ana_. It is expected that with high magnetic field strength and indirect detection of glutamine and glutamate H3 (with the aid of selective ^1^H and/or ^13^C editing to separate glutamate and glutamine H3 signals) further improvements in the determination of the astroglial TCA cycle rate may be possible. Nevertheless, at metabolic and isotopic steady state, the V_cyc_/neuronal V_TCA_ (V_cyc_/^n^V_TCA_) ratio can be reliably measured. This is because the steady state signal of glutamate C3, in this case, results from a balance among the fluxes which feed label from astroglial glutamine C3 via the glutamate–glutamine cycle and outgoing fluxes to the astroglial glutamine C3 and label scrambling to other carbon positions by the neuronal TCA cycle.

A similar measurement of ^13^C label flow from astroglial glutamine to neuronal glutamate can be made by taking advantage of the glial specific substrate acetate (Muir et al., 1986; Waniewski and Martin, 1998). Prolonged incubation of neuron culture with ^13^C-labeled acetate showed no enrichment of ^13^C labels in glutamate, glutamine, or GABA (Sonnewald et al., 1993). Exogenous [2-^13^C] acetate is metabolized via astroglial acetate-CoA ligase and TCA cycle, and subsequently produces [4-^13^C] glutamine. Then, ^13^C labels enter neuronal compartments via the glutamate–glutamine cycling flux to produce [4-^13^C] glutamate. Because of the high specificity of acetate metabolism [2-^13^C] acetate has been used *in vivo* to quantitatively characterize astroglial metabolism in the human brain (Blüml et al., 2002; Lebon et al., 2002). When [1-^13^C] acetate, instead of [2-^13^C] acetate, is administered, the primary route of intercompartmental ^13^C label transfer is from astroglial glutamine C5 to neuronal glutamate C5. Unlike the avid utilization of glucose by brain, the rate of ^13^C label transfer from acetate to glutamine and glutamate is significantly slower, making direct measurement of the absolute rate of metabolic fluxes by *in vivo* MRS more challenging, especially on human subjects. Again, similar to the strategy adopted for probing ^13^C label flow from astroglial glutamine to neuronal glutamate using [2-^13^C] or [2,5-^13^C_2_] glucose, the V_cyc_/^n^V_TCA_ flux ratio can be measured at metabolic and isotopic steady state during [2-^13^C] acetate infusion (Lebon et al., 2002). This is because the steady state signal of glutamate C4 reflects a balance among the fluxes that feed label from astroglial glutamine C4 via the glutamate–glutamine cycle and the outgoing fluxes to the astroglial glutamine C4 and label shift to C3 by the neuronal TCA cycle. A detailed mathematical treatment is given in the section “Effects of glutamine isotopic dilution on the determination of the glutamate–glutamine cycling rate.”

Like acetate, monocarboxylic acids such as β-hydroxybutyrate (bHB) and lactate readily cross the blood brain barrier via the monocarboxylate transporters and are utilized as fuels. Both ^13^C-labeled bHB and lactate have been used to assess ^13^C label incorporation into brain glutamate and glutamine (Kunnecke et al., 1993; Pan et al., 2002; Tyson et al., 2003; Boumezbeur et al., 2010b; Jiang et al., 2011; Xiang and Shen, 2011a). Tissue culture studies of neonatal and embryonic mouse cortex (Lopes-Cardozo et al., 1986) have reported that the majority of the bHB consumed in neurons is oxidized, whereas in astroglial cells it is only 20%. Thus, bHB oxidation should be more indicative of neuronal metabolism than astroglial metabolism. But unlike the high compartmental specificity associated with acetate, oxidation of bHB by both neuronal and astroglial TCA cycles has to be modeled in metabolic pathway analysis. The situation is similar for the case of ^13^C-labeled lactate infusion. Similar to glucose, oxidation of lactate by both neuronal and astroglial TCA cycles needs to be modeled although the majority of plasma lactate is metabolized in neurons (Boumezbeur et al., 2010b).

## Differential equations of the glutamate–glutamine cycle

The ^13^C labeling dynamics implied in the two-compartment model depicted in Figures 2 and 3 can be described explicitly using coupled linear differential equations, following standard theory of chemical kinetics. That is, the rate of change in concentration equals the sum of total incoming fluxes minus the sum of total outgoing fluxes. Here we use the *in vivo* time courses of the [4-^13^C] glutamate and [4-^13^C] glutamine concentrations measured during infusion of [1-^13^C] or [1,6-^13^C_2_] glucose as a simple illustration. The two-compartment metabolic model is shown in Figure 2. As described by the equations below, ^13^C label enters the [4-^13^C] glutamine pool from both neuronal [4-^13^C] glutamate, which is taken up by astroglia, and directly from the astroglial TCA cycle coupled to the anaplerotic pathway.

### Astroglial compartment

Based on metabolic steady-state considerations, the only net pathways of glutamine synthesis (V_Gln_) are the glutamate–glutamine cycle (V_cyc_) and de novo glutamine synthesis by the anaplerotic pathway (V_ana_) (Equation 1). The enrichment of the astroglial [4-^13^C] glutamate pool can be calculated using the standard small pool assumption that the pool size is small enough to be approximated as instantaneously reaching isotopic steady state with the isotopic fluxes passing through the pool. Experimental evidence for this assumption of a relatively small glutamate pool in astrocytes comes from glutamate immunocytochemistry (Ottersen et al., 1992), studies showing that the enrichment in glutamine from infused ^13^N and ^15^N ammonia is several times greater than that of glutamate (Cooper et al., 1979), and estimates based on *in vivo* ^13^C MRS (Lebon et al., 2002). The reversal of the normal product-precursor relationship during ammonia fixation is seen as evidence that the astroglial glutamate pool is much smaller than the glutamine pool and undergoes rapid isotopic turnover. The steady state equation for the concentration of astroglial [4-^13^C]-glutamate ([^a^Glu4^*^]) is given by Shen et al. (1999):

(3) d[aGlu4∗]/dt=VaTCA[Lac3∗]/[Lac]+Vcyc[nGlu4∗]/[nGlu]−(Vcyc+Vana)[aGlu4∗]/[aGlu]−(aVTCA−Vana)[aGlu4∗]/[aGlu]=0

where ^a^V_TCA_ is the astroglial tricarboxylic acid cycle flux, V_cyc_ is the glutamate–glutamine cycle flux, and V_ana_ is the anaplerotic flux. [Lac3^*^]/[Lac] denotes the fractional enrichment of the label feeding flux at pyruvate dehydrogenase in astrocytes (The exchange between lactate and pyruvate catalyzed by lactate dehydrogenase is much faster than the rate of the pyruvate dehydrogenase reaction.). [^n^Glu4^*^]/[^n^Glu] denotes the fractional enrichment of the neuronal glutamate pool. The labeling of astroglial [4-^13^C] glutamine is described by

(4) $$\text{d}{[}^{\text{a}}\text{Gln}4^{*}]/\text{dt}={\text{V}}_{\text{Gln}}{[}^{\text{a}}\text{Glu}4^{*}]/{[}^{\text{a}}\text{Glu}]-{\text{V}}_{\text{cyc}}{[}^{\text{a}}\text{Gln}4^{*}]/{[}^{\text{a}}\text{Gln}]-({\text{V}}_{\text{efflux}}+{\text{V}}_{\text{dil}(\text{Gln})}){[}^{\text{a}}\text{Gln}4^{*}]/{[}^{\text{a}}\text{Gln}]$$

where V_Gln_ is the total glutamine synthesis rate, V_efflux_ is the net glutamine efflux rate, and V_dil(Gln)_ is the flux term that accounts for the additional isotopic dilution of glutamine observed experimentally (vide infra).

### Neuronal compartment

The labeling of the neuronal [4-^13^C] glutamine pool can be described by the same small pool isotopic steady-state assumption:

(5) $$\text{d}{[}^{\text{n}}\text{Gln}4^{*}]/\text{dt}={\text{V}}_{\text{cyc}}({[}^{\text{a}}\text{Gln}4^{*}]/{[}^{\text{a}}\text{Gln}]-{[}^{\text{n}}\text{Gln}4^{*}]/{[}^{\text{n}}\text{Gln}])=0$$

The ^13^C labeling kinetics of the neuronal glutamate pool is given by

(6) $$\text{d}{[}^{\text{n}}\text{Glu}4^{*}]/\text{dt}{=}^{\text{n}}{\text{V}}_{\text{TCA}}[\text{Lac}3^{*}]/[\text{Lac}]+{\text{V}}_{\text{cyc}}{[}^{\text{n}}\text{Gln}4^{*}]/{[}^{\text{n}}\text{Gln}]-({\text{V}}_{\text{cyc}}{+}^{\text{n}}{\text{V}}_{\text{TCA}}){[}^{\text{n}}\text{Glu}4^{*}]/{[}^{\text{n}}\text{Glu}]$$

where ^n^V_TCA_ is the neuronal tricarboxylic acid cycle flux. If the input plasma glucose concentration and [1-^13^C] or [1,6-^13^C_2_] glucose fractional enrichment time courses can be described using simple analytical functions the coupled differential equations (3–6) can be solved exactly using either inverse Laplace transform or eigen value decomposition. Since the real arterial input function is time-varying and coupled to glucose transport kinetics numerical, iterative methods are used in practice to derive V_cyc_ by minimizing the least-square difference between the calculated and measured values for [Glu4^*^] and [Gln4^*^]. The time courses of glutamate and glutamine C3 (and C2) measured during infusion of [1-^13^C] or [1,6-^13^C_2_] glucose can be described mathematically using the same principle. Similarly, the time courses of ^13^C-labeled glutamate and glutamine measured using other labeling strategies can also be modeled to extract metabolic fluxes of interest.

Instead of attempting to summarize the full sets of differential equations applicable to individual labeling strategies we examine the differential equations for the simplified two-compartment model depicted in Figure 3, which captures the main features of the full two-compartment model shown in Figure 2. In particular, the effects of isotopic dilution of glutamine on various labeling strategies for determining the glutamate–glutamine cycling rate are illustrated using the simplified two-compartment model.

## Origins of glutamine isotopic dilution

Although the complete TCA cycle is known to occur exclusively in mitochondria many enzymes involved in the TCA cycle in brain (e.g., malate dehydrogenase, aconitase, and isocitrate dehydrogenase) are found in the cytosol of neurons and astrocytes as well (Koen and Goodman, 1969; Siesjo, 1978; Rodrigues and Cerdán, 2006). Only brain pyruvate dehydrogenase, citrate synthase, fumarase, succinate dehydrogenase and succinate thiokinase are exclusively mitochondrial (e.g., Siesjo, 1978; Akiba et al., 1984; Lai et al., 1989; Rodrigues and Cerdán, 2006). Many TCA cycle intermediates outside the mitochondria are in exchange with their mitochondrial counterparts via various transporters and channels. In terms of metabolic modeling the above processes contribute to “isotopic dilution” because a net loss of ^13^C labels occurs via exchange with unlabeled cytosolic pools which are connected to non-TCA cycle pathways. In addition, the net oxidation of (unlabeled) non-glucose fuels in brain is significant despite that glucose is the major source of carbon oxidized in the TCA cycles of brain cells under normal physiological conditions. As described in “Labeling strategies” the contributions of several non-glucose fuels to brain metabolism have been probed by administering the fuel source labeled with ^13^C. Despite the inflows of unlabeled substrates, the TCA cycle rate can still be correctly determined by incorporating isotopic dilution pathways in the metabolic models. The experimentally determined fractional enrichment of glutamate C4 was smaller than that expected with [1-^13^C] or [1,6-^13^C_2_] glucose being the sole carbon source for glutamate. This dilution includes contributions from the influx of unlabeled substrates from blood and cytosol, including lactate, pyruvate, ketone bodies, free amino acids, as well as amino acids produced by protein degradation. On the basis of a quantitative analysis of various carbon flows into brain reported in the literature, lactate was considered to be the major contribution to this dilution under normal physiologic conditions (Mason et al., 1995). Hence, V_dil(Lac)_ has been used to represent the lumped effect of these dilution fluxes.

In addition to the isotopic dilution effect observed at glutamate, astroglial dilution flux accounts for an additional 26% of label dilution at glutamine C4 (Shen et al., 1999). A detailed analysis of glutamate and glutamine dilutions has been given by Dienel and Cruz (2009). There are several potential sources of this dilution although it is unclear if any single source dominates. Astroglial glutamine is in exchange with unlabeled glutamine in blood across the blood–brain barrier (V_efflux_, see Figure 1), primarily mediated by N-system transporters (Bröer and Brookes, 2001). Oxidation of short- and medium-chain free fatty acids (e.g., acetate and bHB) occur significantly in the astroglia with acetate regarded as an astroglial specific substrate. ^14^C-acetate (Cruz et al., 2005) and ^13^C-octanoate (Ebert et al., 2003) studies showed that these endogenous free fatty acids at basal blood levels contribute significantly to astroglial oxidation (see Table 2). For example, Cruz et al. (2005) found that, depending on brain region and level of activity, acetate utilization may provide 28–115% of total astroglial oxidation. Oxidation of these free fatty acids undoubtably generates unlabeled acetyl-CoA leading to additional label dilution at glutamine C4. Branched chain amino acids readily cross the blood–brain barrier and can act as a fuel source in the brain (Hutson et al., 2005). [U-^14^C] leucine injected systemically was found to label brain glutamine, glutamate, and aspartate in early neurochemical research (Patel and Balázs, 1970). Therefore, branched chain amino acids metabolized to acetyl-CoA or glutamate also contribute to isotopic dilution of glutamine C4. As a result of the additional isotopic dilution in astroglial cells, the fractional enrichment of glutamine C4 is significantly lower than glutamate C4 at all times during [1-^13^C] or [1,6-^13^C_2_] glucose infusion, including the isotopic steady state. Similar to the case of V_dil(Lac),_ we use a single flux term V_dil(Gln)_ to represent the lumped glutamine dilution fluxes, including dilutions at both glutamine and acetyl-CoA levels.

**Table 2:** ^13^C labeling strategies for studying the glutamate–glutamine cycle^*^

**GLUCOSE**	
[1-^13^C] or [1,6-^13^C_2_] glucose	Primary route of label transfer: glutamate C4 → glutamine C4
	Anaplerotic pathway: glutamine C2 → glutamate C2
[2-^13^C] or [2,5-^13^C_2_] glucose	Primary route of label transfer: glutamate C5 → glutamine C5
	Anaplerotic pathway: glutamine C3 → glutamate C3
ACETATE^**^	
[2-^13^C]acetate	Primary route of label transfer: glutamine C4 → glutamate C4
[1-^13^C]acetate	Primary route of label transfer: glutamine C5 → glutamate C5
**bHB AND LACTATE**	
[2,4-^13^C_2_]bHB, [3-^13^C] lactate	Primary route of label transfer: glutamate C4 → glutamine C4
[1,3-^13^C_2_]bHB, [ 2-^13^C] lactate	Primary route of label transfer: glutamate C5 → glutamine C5

* For simplicity, only intercompartmental label transfer during the first turn of the TCA cycles is shown.

** Isotope labels originating from ^13^C-ethanol enter the brain after being converted into acetate in the liver. The labeling pathway for ^13^C-labeled ethanol is the same as that of acetate (Xiang and Shen, 2011b).

## Effects of glutamine isotopic dilution on the determination of the glutamate–glutamine cycling rate

The extraction of V_cyc_ requires metabolic modeling by least square minimization. In least square minimization, the cost function is proportional to χ^2^ when error per data point has the same noise level (von Mises, 1964). In metabolic modeling fluxes are determined by minimizing χ^2^. Generally, the terms in χ^2^ are not all statistically independent. For non-linear systems, such as the two-compartment model of the glutamate–glutamine cycle the analytical derivation of the probability density function for different χ^2^ at its minimum is very difficult. Even for the simplified two-compartment model analytical derivation of the probability density function for V_cyc_ remains a daunting task. Instead, Monte Carlo simulation can be used to accurately assess the reliability of V_cyc_. Using the Monte Carlo method, turnover data sets drawn from the predefined metabolic model are numerically synthesized. Subsequently, the synthesized data are used to determine both the probability density function of the χ^2^-statistic, and the reproducibility of metabolic fluxes extracted using the fitting procedure. To ensure that the optimization method of choice works properly for metabolic modeling statistical analysis of χ^2^ data can be performed. When the number of noise realizations is sufficiently large, the χ^2^-statistic should be normally distributed with a mean value of *N–n* and a standard deviation of sqrt[2(*N*–*n*)] for a small set of metabolic fluxes. Here *N* is the total number of data points in the turnover curves and *n* the number of free metabolic fluxes to be derived from the fit (von Mises, 1964). Therefore, the mean and standard deviation of χ^2^ are indicators of the goodness-of-fit in Monte Carlo analysis. When metabolic fluxes are derived based on local instead of global minima, significant deviations from the theoretical χ^2^-statistic are expected.

### [1-^13^C] or [1,6-^13^C_2_] glucose infusion

The effects of glutamine isotopic dilution on determination of the glutamate–glutamine cycling rate depend on specific labeling strategies. For studies utilizing infusion of [1-^13^C] or [1,6-^13^C_2_] glucose, which produce the maximum MRS signal-to-noise ratio with direct or indirect detection of aliphatic carbons, explicit incorporation of the glutamine isotopic dilution flux into the two-compartment glutamate–glutamine cycling model is critical. Without the astroglial glutamine dilution flux, the isotopic steady state fractional enrichment of the downstream glutamine C4 would have been the same as that of glutamate C4. The experimentally measured glutamine C4 turnover curve results from a balance between incoming ^13^C labeled flux entering the glutamine pool (e.g., the glutamate–glutamine cycling flux) and outgoing ^13^C labeled flux leaving the glutamine pool (e.g., glutamate–glutamine cycling flux and the astroglial dilution flux). The effects of glutamine isotopic dilution on determination of the glutamate–glutamine cycling rate are best illustrated using the simplified two-compartment model depicted in Figure 3, which represents the dominant metabolic pathway for the trafficking of ^13^C labels in brain between neurons and astroglia. For this simplified model, the differential equations (4–6) describing the kinetics of ^13^C label incorporation into glutamine C4 are simplified into:

(7) $$\text{d}{[}^{\text{a}}\text{Gln}4^{*}]/\text{dt}={\text{V}}_{\text{cyc}}{[}^{\text{n}}\text{Glu}4^{*}]/{[}^{\text{n}}\text{Glu}]-{\text{V}}_{\text{cyc}}{[}^{\text{a}}\text{Gln}4^{*}]/{[}^{\text{a}}\text{Gln}]-{\text{V}}_{\text{dil}(\text{Gln})}{[}^{\text{a}}\text{Gln}4^{*}]/{[}^{\text{a}}\text{Gln}]$$

Note that the above Equation (7) is very similar to the full differential equation describing the labeling of astroglial glutamine C4 (Equation 4). To arrive at Equation (7), the term V_efflux_([^a^Glu4^*^]/[^a^Glu] – [^a^Gln4^*^]/[^a^Gln]) in Equation (4) was omitted. Since the astroglial glutamate pool size is much smaller than the astroglial glutamine pool size, approximately, we have [^a^Glu4^*^]/[^a^Glu] ≈ [^a^Gln4^*^]/[^a^Gln], which reduces Equation (4) to Equation (7). Here we have again applied the standard small pool approximation although a more accurate description of the fractional enrichment of the astroglial glutamate pool is given by Equation (3). Note that the incoming astroglial dilution flux involves ^12^C-labeled Gln C4, which does not enter into Equation (7).

At isotopic steady state, the following is obtained from Equation (7):

(8) $${[}^{\text{a}}\text{Gln}4^{*}]/{[}^{\text{a}}\text{Gln}]=({[}^{\text{n}}\text{Glu}4^{*}]/{[}^{\text{n}}\text{Glu}]){\text{V}}_{\text{cyc}}/({\text{V}}_{\text{cyc}}+{\text{V}}_{\text{dil}(\text{Gln})})$$

Note that, if V_dil(Gln)_ is incorrectly set to zero, the fractional enrichment of the down-stream glutamine C4 approaches that of the upstream glutamate C4 after the initial glucose infusion period, progressively losing sensitivity to the label-feeding flux (V_cyc_) over time and causing an overall reduced sensitivity to V_cyc_. At isotopic steady state, the fractional enrichments of glutamine C4 and glutamate C4 become identical if V_dil(Gln)_ = 0. That is, at isotopic steady state the glutamine C4 curve is independent of V_cyc_. In contrast, when the V_dil(Gln)_ term is included in the differential equation (7) indicates that the whole glutamine C4 turnover curve is sensitive to V_cyc_. This sensitivity is not lost at isotopic steady state. Unless V_cyc_ >> V_dil(Gln)_, the fractional enrichment of glutamate C4 will always be larger than the fractional enrichment of glutamine C4 when [1-^13^C] or [1,6-^13^C_2_] glucose is infused. As shown by Equations (7) and (8), V_dil(Gln)_ and V_cyc_ have different effects on the turnover of glutamine C4, allowing the two fluxes to be separately determined.

**Figure 4:**
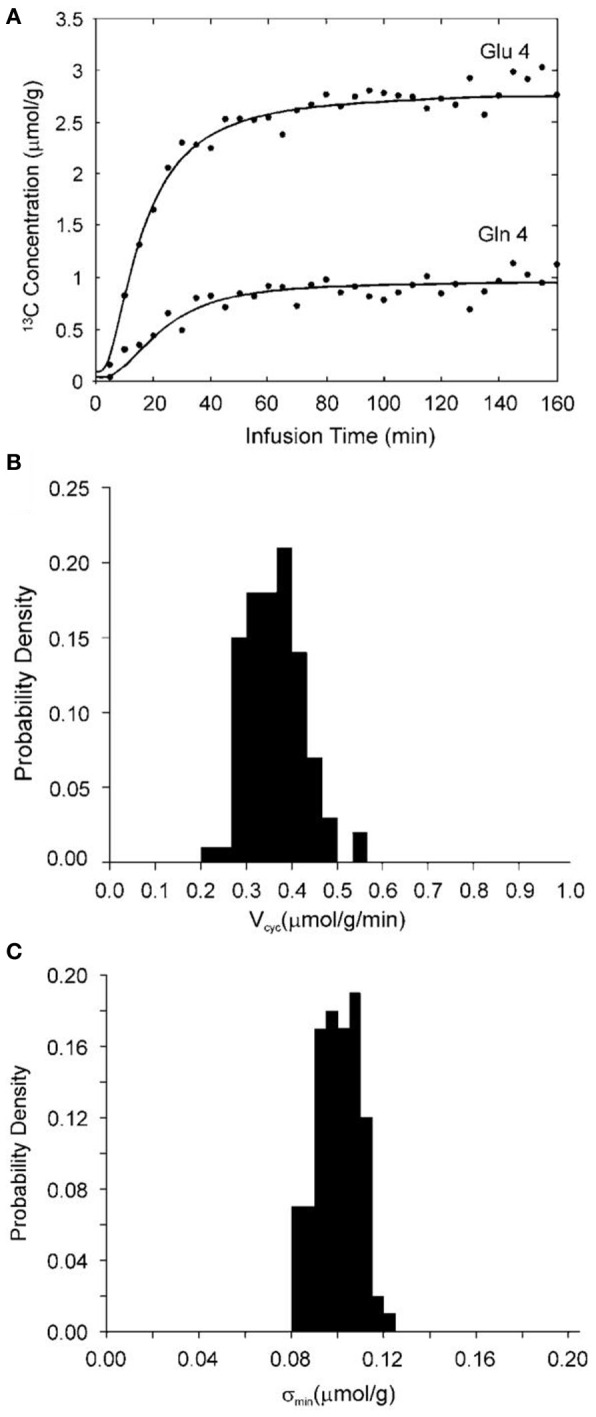
**(A)** Synthetic glutamate C4 and glutamine C4 turnover curves (lines) generated by solving the four coupled differential equations describing the model in Figure 2 with [1-^13^C] or [1,6-^13^C_2_] glucose infusion, the mean metabolic fluxes, and adding normally distributed noise (dots). The astroglial dilution is revealed in **(A)** by comparing the fractional enrichments of glutamate and glutamine C4. At the end point of glucose infusion, the concentration of glutamine and glutamate with ^13^C labels at C4 is 0.96 and 2.75 μmol/g, respectively. The end point fractional enrichment of glutamine C4 is 26% lower than that of glutamate C4 as observed experimentally (Shen et al., 1999). **(B)** An example of the probability density function for V_cyc_ with constraints ^a^V_TCA_ ≤ 0.1, V_dil(Lac)_ = 0.05, and V_dil(Gln)_ = 0.14 μmol/g/min. **(C)** The corresponding probability density function of the “noise level” after fitting (σ_min_). The mean χ^2^ obtained is 60.8 with a standard deviation of 11.2. σ_min_ averaged over the 100 Monte Carlo data sets is 0.098 μmol/g with a standard deviation of 0.009 μmol/g. (This image is reproduced from Shen et al., 2009).

The above analysis was validated by the use of the full two-compartment model shown in Figure 2, the corresponding full differential equations (3–6), and Monte Carlo simulation (Shen et al., 2009). Specifically, glutamate C4 and glutamine C4 turnover curves (see Figure 4A) were generated by solving the differential equations (3–6) describing the full two-compartment model with the mean metabolic fluxes obtained from Shen et al. (1999). The total infusion time is 160 min with 32 data points per curve. Gaussian white noise with standard deviation σ = 0.1 μmol/g was added to assess the accuracy and reproducibility of V_cyc_. [1-^13^C] or [1,6-^13^C_2_] glucose infusion was assumed. At the end point of glucose infusion the fractional enrichment of glutamine C4 is 26% lower than that of glutamate C4 as observed experimentally (Shen et al., 1999). This data set was fitted with the two-compartment model with suitable constraints using the simulated annealing method which is well-known for its robustness in determining global minimum in a multidimensional error space. Metabolic fluxes were determined by minimizing the summed square cost function which is proportional to χ^2^. The above procedure was repeated 100 times with the same noise variance but with different noise realizations. A probability density function for V_cyc_ is shown in Figure 4B. For the data set shown in Figure 4A the mean χ^2^ obtained with 100 noise realizations is 60.8 with a standard deviation of 11.2. Comparison with the theoretical mean χ^2^ of 32 × 2 − 4 = 60 and standard deviation of 11.0 (the theoretical values are obtained when the number of noise realizations approaches infinity) indicates that global minima were found using the simulated annealing method. The χ^2^ data were also used to calculate the noise level after minimization by simulated annealing (σ_min_). In the above case, σ_min_ averaged across the 100 simulations is 0.098 μmol/g with a standard deviation of 0.009 μmol/g. The corresponding probability density function of σ_min_ is shown in Figure 4C. Because poor data fitting caused by false minima was avoided by the use of simulated annealing the average σ_min_ is expected to be slightly less than the added noise level (σ) with a relative standard deviation of slightly less than 10%. This is exactly what was found. This result also confirmed that the constraints imposed on the metabolic fluxes did not alter the quality of the fit.

The importance of the glutamine dilution flux in the accuracy and precision of determining V_cyc_ was also assessed using numerical Monte Carlo simulations with V_dil(Gln)_ forced to zero. When V_dil(Gln)_ is set to zero the ^13^C-labeled glutamine concentration predicted by the model shown in Figure 2 is higher than that shown in Figure 4A. To match the concentration of ^13^C-labeled glutamine in Figure 4A and the ^13^C-labeled glutamine concentration with V_dil(Gln)_ = 0, the total glutamine concentration needs to be reduced to 2.8 mmol/L in the Monte Carlo simulations because the actual ^13^C fractional enrichment of glutamine C4 is lower than that of glutamate C4. For a noise level of σ = 0.2 μmol/g, a relative standard deviation of 438% was calculated for V_cyc_, indicating this flux cannot be reliably determined under the simulated condition [V_dil(Gln)_ = 0] using the two-compartment model shown in Figure 2. At the noise level of σ = 0.1 μmol/g the relative standard deviation of the calculated V_cyc_ is 66%. When ^a^V_TCA_ was constrained not to exceed 0.1 μmol/g/min and simultaneously V_efflux_ was forced to 0.2^*^V_Gln_ a large reduction in the uncertainty of V_cyc_ was found. In all cases investigated using numerical Monte Carlo simulations a smaller standard deviation was found when the V_dil(Gln)_ term was included in the modeling.

### [2-^13^C] or [2,5-^13^C_2_] glucose infusion

The effects of glutamine isotopic dilution on determination of the glutamate–glutamine cycling rate using [2-^13^C] or [2,5-^13^C_2_] glucose infusion are expected to be quite different. Glucose labeled at [2-^13^C] or [2,5-^13^C_2_] is metabolized through glycolysis to [2−^13^C] pyruvate, which enters the TCA cycle through one of two different pathways. Entry of [2-^13^C] pyruvate through pyruvate dehydrogenase leads to labeling of glutamate (and glutamine) at C5, whereas entry through pyruvate carboxylase (anaplerosis) leads to labeling of glutamate (and glutamine) at C3 in the first turn of the TCA cycle followed by C2 and C1 in subsequent turns. Similar to [1-^13^C] or [1,6-^13^C_2_] glucose infusion, potential label scrambling at fumarate during [2-^13^C] or [2,5-^13^C_2_] glucose infusion labels glutamate and glutamine C2 in the first turn of the TCA cycle (see the “Labeling strategies” section). Half of the ^13^C labels on glutamate C3 move to glutamate C2 due to symmetric label scrambling of the TCA cycle. ^13^C-labeled glutamate C3 is also in exchange with ^13^C-labeled glutamine C3 via the glutamate–glutamine cycle. Using the simplified model shown in Figure 3 the following differential equation describes the kinetics of ^13^C-labeled glutamate C3:

(9) $$\text{d}{[}^{\text{n}}\text{Glu}3^{*}]/\text{dt}=-0.5^{\text{n}}{\text{V}}_{\text{TCA}}{[}^{\text{n}}\text{Glu}3^{*}]/{[}^{\text{n}}\text{Glu}]+{\text{V}}_{\text{cyc}}({[}^{\text{a}}\text{Gln}3^{*}]/{[}^{\text{a}}\text{Gln}]-{[}^{\text{n}}\text{Glu}3^{*}]/{[}^{\text{n}}\text{Glu}])$$

At metabolic and isotopic steady state, Equation (9) becomes:

(10) Vcyc/VnTCA=0.5∗([nGlu3∗]/[nGlu])/([aGln3∗]/[aGln]−[nGlu3∗]/[nGlu])

by setting d[^n^Glu3^*^]/dt to zero. Equation (10) is identical to Equation (4) in Sibson et al. (2001) if a small correction term ([^n^Glu4^*^]/[^n^Glu]) is ignored. Equation (4) in Sibson et al. (2001) was derived using the full two-compartment model (Figure 2). Note that, because Equation (9) describes the kinetics of a predominantly neuronal signal ([^n^Glu3^*^]), the astroglial V_dil(Gln)_ term does not appear in Equations (9) or (10).

To describe the labeling kinetics of glutamine C3 the main label-feeding flux via the astroglial anaplerotic pathway needs to be incorporated. The following equation is then obtained:

(11) $$\text{d}{[}^{\text{a}}\text{Gln}3^{*}]/\text{dt}={\text{V}}_{\text{ana}}[\text{Lac}2^{*}]/[\text{Lac}]+{\text{V}}_{\text{cyc}}({[}^{\text{n}}\text{Glu}3^{*}]/{[}^{\text{n}}\text{Glu}]-{[}^{\text{a}}\text{Gln}3^{*}]/{[}^{\text{a}}\text{Gln}])-{\text{V}}_{\text{dil}(\text{Gln})}{[}^{\text{a}}\text{Gln}3^{*}]/{[}^{\text{a}}\text{Gln}]$$

Equation (11) is analogous to Equation (7) for [1-^13^C] or [1,6-^13^C_2_] glucose infusion. At metabolic and isotopic steady state Equation (11) is reduced to:

(12) $${\text{V}}_{\text{dil}(\text{Gln})}/{\text{V}}_{\text{cyc}}=({\text{V}}_{\text{ana}}/{\text{V}}_{\text{cyc}})\times ([\text{Lac}2^{*}]/[\text{Lac}])/({[}^{\text{a}}\text{Gln}3^{*}]/{[}^{\text{a}}\text{Gln}])+({[}^{\text{n}}\text{Glu}3^{*}]/{[}^{\text{n}}\text{Glu}])/({[}^{\text{a}}\text{Gln}3^{*}]/{[}^{\text{a}}\text{Gln}])-1$$

The most interesting conclusion of the above analysis of the effects of astroglial isotopic dilution of glutamine on determining V_cyc_ using [2-^13^C] or [2,5-^13^C_2_] glucose infusion is that V_cyc_ (or V_cyc_/^n^V_TCA_ if only steady state fractional enrichments of glutamate and glutamine C3 are measured) is insensitive to astroglial isotopic dilution of glutamine. As described in “Labeling strategies,” the sensitivity of the [2-^13^C] or [2,5-^13^C_2_] glucose infusion with detection of aliphatic carbons is limited because of the small V_ana_ flux. Dynamic studies necessary for determining the absolute V_cyc_ flux may be attempted by boosting sensitivity with high magnetic fields and indirect proton detection (with ^13^C or proton editing to separate spectral overlap between glutamate H3 and glutamine H3). An alternative experimental strategy also exists. Recent MRS data of human brain acquired during [2-^13^C] glucose infusion have shown that the sensitivity of carboxyl/amide carbon detection is sufficient for the reliable measurement of glutamate turnover at 3 Tesla (Li et al., 2009). This would allow determination of the absolute, predominantly neuronal TCA cycle rate. Since the recycle delay of the carboxyl/amide carbon MRS experiment is relatively long, interleaved detection of the aliphatic glutamate C3 and glutamine C3 signals can be performed in principle. Then, by adding all the time course data of glutamate C3 and glutamine C3, the V_cyc_/^n^V_TCA_ ratio can be simultaneously measured [by utilizing the integrated form of Equation (9)]. By combining data from carboxyl/amide and aliphatic spectral regions, V_cyc_ can be determined independent of V_dil(Gln)_, in contrast to the case of [1-^13^C] or [1,6-^13^C_2_] glucose infusion.

### [2-^13^C] acetate infusion

The insensitivity to astroglial glutamine dilution flux V_dil(Gln)_ in the determination of V_cyc_ by the use of [2-^13^C] or [2,5-^13^C_2_] glucose infusion is a direct result of extracting V_cyc_ from signals (d[^n^Glu3^*^]/dt) of the neuronal compartment (see Equation 9). In [2-^13^C] or [2,5-^13^C_2_] glucose infusion experiments the flux causing the increased labeling of glutamate C3 signal comes from glutamine C3 predominantly located in the astroglia. A similar scenario can be generated by the use of [2-^13^C] acetate infusion. During [2-^13^C] acetate infusion, ^13^C label from [4-^13^C] glutamine enters neuronal compartments via the glutamate–glutamine cycle to produce [4-^13^C] glutamate. Here the glutamate C4 plays the same role as that of glutamate C3 during [2-^13^C] or [2,5-^13^C_2_] glucose infusion. The corresponding differential equation describing the labeling kinetics of glutamate C4 is:

(13) $$\text{d}{[}^{\text{n}}\text{Glu}4^{*}]/\text{dt}={-}^{\text{n}}{\text{V}}_{\text{TCA}}{[}^{\text{n}}\text{Glu}4^{*}]/{[}^{\text{n}}\text{Glu}]+{\text{V}}_{\text{cyc}}({[}^{\text{a}}\text{Gln}4^{*}]/{[}^{\text{a}}\text{Gln}]-{[}^{\text{n}}\text{Glu}4^{*}]/{[}^{\text{n}}\text{Glu}])$$

for the simplified two-compartment model shown in Figure 3. Essentially the same equation is obtained when the full two-compartment model (Figure 2) was used (Lebon et al., 2002). Similar to Equation (9), V_cyc_, in principle, can be determined from the ^13^C MRS turnover data. The sensitivity of the acetate infusion experiment, however, is only a fraction of that of the corresponding [1-^13^C] or [1,6-^13^C_2_] glucose infusion experiment. As an alternative to dynamic studies, the V_cyc_/^n^V_TCA_ flux ratio can be determined from spectra acquired at the metabolic and isotopic steady state where d[^n^Glu4^*^]/dt = 0:

(14) Vcyc/VnTCA=([nGlu4∗]/[nGlu])/([aGln4∗]/[aGln]−[nGlu4∗]/[nGlu])

Note that, as expected, the astroglial glutamine dilution flux V_dil(Gln)_ does not appear in either Equations (13) or (14). Therefore, V_cyc_ determined in this manner is also insensitive to V_dil(Gln)_.

As well-known in statistics, over-parameterization or over-fitting of a model leads to increased uncertainty. A familiar example is spectral fitting in quantification of crowded proton MRS spectra. When the spectral model is over-parameterized, erroneous concentration and increased uncertainty are easily obtained. In the case of [2-^13^C] or [2,5-^13^C_2_] glucose and [2-^13^C] acetate infusion experiments described here, the addition of flux terms [e.g., V_dil(Gln)_] to which the data are insensitive (Shestov et al., 2012) can lead to increased uncertainly in V_cyc_.

To extract the absolute flux of the glutamate–glutamine cycle an independent or simultaneous measurement of V_TCA_ needs to be performed. In a recent study, difference in brain mitochondrial metabolism of young and old healthy human subjects was studied by infusing [1-^13^C] glucose in one experiment and [2-^13^C] acetate to the same subject in a separate experiment (Boumezbeur et al., 2010a). For [2-^13^C] acetate infusion experiments, it is possible to infuse [2-^13^C] acetate and [2-^13^C] (or [2,5-^13^C_2_]) glucose simultaneously and measure V_TCA_ in the carboxyl/amide spectral region and measure the V_cyc_/^n^V_TCA_ flux ratio in the aliphatic spectral region as outlined for the case of [2-^13^C] or [2,5-^13^C_2_] glucose infusion.

*In vivo* double-labeling experiments have been performed recently on both animals (Deelchand et al., 2009; Xiang and Shen, 2011a) and human subjects (Li et al., 2012). Since carboxyl/amide carbons are located at the end of the carbon skeleton of amino acids they can form either a singlet if its neighbor carbon is ^12^C, or a doublet if its neighbor carbon is ^13^C, leading to natural simplification of their isotopomer patterns (Xiang and Shen, 2011a). When ^13^C-labeled glucose is administered the glutamate and glutamine C4-C5 moieties are labeled by glucose C1-C2 and/or C5-C6, regardless of the number of the turns of the TCA cycle. Consequently, when the input is [1,2-^13^C_2_] acetyl-CoA, glutamate C5 and glutamine C5 can only form doublet signals. Vice versa, when the input is [2-^13^C] acetyl-CoA glutamate C5 and glutamine C5 can only form singlet signals. This unique feature of the carboxyl/amide carbons makes it possible to simultaneously and unambiguously detect the labeling of glutamate and glutamine by two substrates with the second substrate (e.g., acetate) producing singlets only. Because of the large one-bond ^13^C-^13^C homonuclear J coupling between a carboxyl/amide carbon and an aliphatic carbon (~50 Hz), the singlet and doublet signals of the same carboxyl/amide carbon can be well distinguished. It is readily conceivable that one may use the glutamate C5 doublet generated from uniformly labeled glucose to measure the absolute flux of V_TCA_ and the glutamate C5 and glutamine C5 singlets generated from [1-^13^C] acetate to measure the V_cyc_/^n^V_TCA_ flux ratio. V_cyc_ measured in this manner is expected to be insensitive to the astroglial glutamine dilution flux based on the above analysis.

## Conclusion

*In vivo* ^13^C MRS has evolved into a major non-invasive tool for assessing glutamatergic function in both human subjects and animal models. Recent development of ^13^C MRS techniques has been spurred by renewed interest in the glutamate–glutamine cycle proposed decades ago. Like all metabolic models the two-compartment glutamate–glutamine cycling model has generated controversy. While the dual roles of glutamate as primary excitatory neurotransmitter in the CNS and as a key metabolite linking carbon and nitrogen metabolism make it possible to probe glutamate neurotransmitter cycling using MRS this dual role also requires one to separate neurotransmission events from metabolism. In spite of the on-going controversies, the *in vivo* MRS community has made major contributions to our understanding of brain energy consumption and the relationship between neuroenergetics and neurotransmission. Over the past few years our understanding of the neuronal-astroglial two-compartment metabolic model of the glutamate–glutamine cycle has been further advanced. In particular, the importance of isotopic dilution of glutamine in determining the glutamate–glutamine cycling rate using [1-^13^C] or [1,6-^13^C_2_] glucose has been demonstrated and reproduced. It is hoped that the analysis of the astroglial dilution effects and V_dil(Gln)_-insensitive experimental methods for absolute quantification of the glutamate–glutamine cycling rate discussed here will further advance the field. With the increasing availability of high-field MR magnets (7 Tesla or higher) and further developments of MRS techniques characterization of glutamatergic function by *in vivo* ^13^C MRS has the potential to significantly impact both basic and clinical research in neurological and psychiatric disorders.

### Conflict of interest statement

The author declares that the research was conducted in the absence of any commercial or financial relationships that could be construed as a potential conflict of interest.
